# Evaluating Care in Patients with Delirium and Dementia in a Busy District General Hospital: An Audit

**DOI:** 10.1192/bjo.2025.10600

**Published:** 2025-06-20

**Authors:** Muhammad Bilal Saleem, Nataliia Vilinska, Susan Hay

**Affiliations:** 1 Barts Health NHS Trust, London, United Kingdom; 2 North East London Foundation Trust, London, United Kingdom

## Abstract

**Aims:** Delirium is defined as the acute confusional state common in the elderly patient population of hospitals. Alongside existing diagnosed and undiagnosed dementia, it is a common cause of cognitive impairment in the elderly. This project aimed to evaluate the care patients with delirium and dementia received by analysing interventions made by both the referring party and the liaison team as per the local and national guidelines.

**Methods:** The retrospective audit included 39 referrals made to the psychiatric liaison service (PLS) for delirium and/or dementia over three months in patients aged 65 and older. The data was collected from electronic health records to assess parameters such as diagnostic tools used (e.g., ‘4AT Rapid Clinical Test for Delirium’, ‘PINCH ME’ a mnemonic for delirium risk factors including Pain, Infection, Nutrition, Constipation, Hydration, Medication, Environment), cognitive testing, medication reviews, and team actions.

**Results:** This study found ongoing positive practices and areas for improvement in diagnosing and managing delirium and dementia in older adults. Among the 39 patients, 43.6% had a pre-existing diagnosis of dementia, and 28.2% were admitted with acute confusion. While diagnostic blood work, medication reviews, and collateral histories were frequently performed by the referring team, only 51.3% of patients were referred to the Delirium and Dementia (DAD) team. The use of the ‘PINCH ME’ mnemonic was limited, with just 28.2% of cases incorporating it.

After referral, most patients had a history taken, a mental state examination (MSE) conducted, and collateral information gathered by PLS; however, only 10.2% of cases included the use of the mnemonic. Antipsychotics were prescribed in 30.7% of cases. At the point of discharge, 82.1% of cases had follow-up arranged by PLS, with 35% of patients referred to memory clinics for continued care.

**Conclusion:** This audit reveals areas for improvements in the assessment and management of delirium and dementia in hospitalized older adults. Recommendations have been made based on this data to help improve use of 4AT, PINCHME mnemonic and referral process.

Educational initiatives and increased collaboration with the Delirium and Dementia team have been introduced to improve early recognition, standardize care, and align practices with current guidelines. Further study will be conducted to explore its effect as part of a quality improvement project.

